# Medical Morphomaniacs

**Published:** 1910-05-21

**Authors:** 


### Medical Morphomaniacs.

A few months ago Dr. Oscar Jennings—himself a cured morphia *habitué*—committed himself to the assertion that one medical man in four is a drug-taker, most often a morphinist. The statement was met with numerous expressions of incredulity; but the author now returns to the charge in the *British Journal of Inebriety*, and reiterates his conviction with an explanation of the reasons which have led him to it. It cannot be said that he adduces anything whatsoever in the nature of evidence or proof of these generalisations; but he is entitled, as he remarks, to hold his opinions, which are necessarily hard to disprove. In the course of his remarks he refers to the interest which the English doctor, as far as his opportunities permit, takes in golf, riding, and other sports. This has been quoted, and we think rightly, against the author as tending to show that morphinism is less common than he supposes among medical men; for it appears reasonable to suppose that the cool head and steady hands required for such exercises are not likely to be at the command of a drug-slave. But Dr. Jennings will not admit this; he says that even so arduous a pastime as aeroplaning is not incompatible, to his own knowledge, with the taking of morphia or opium, and that the dosing of athletes with strychnine, caffeine, and cocaine is an admitted fact. Such a statement is surely erroneous. It is absurd, he says, to argue that apparently perfect physical and moral health is proof positive of the non-existence of the morphia habit. Here he cuts away from beneath him the main foundations of his crusade. For if all this is to be admitted, then such persons are in no need of reforming their habits and should not be spoken of with opprobrium; in this case Dr. Jennings' own voluntary renunciation of the habit becomes a much less praiseworthy effort than he is still generally credited with. It emerges, however, towards the end of his communication that the author's ideas are not in fact derived from British experience at all, but from reports in France and America; he assumes, he says, that the English are in this respect neither better nor worse than their neighbours. We are far from accepting all his statements about the medical profession in the countries referred to, but in any event it is quite probable that the English doctor leads a healthier life on the whole, and is less likely to fall a victim to drug habits, than his *confrères* abroad, and certainly it cannot be admitted that the large assumption upon which Dr. Jennings' case is founded is in the least legitimate or permissible. The statistics collected among 25 large cities in the United States showed that whereas among criminals 15 per cent. and among prostitutes 20 per cent. take opium or morphine, the proportion among physicians is but 2 per cent., among nurses 1.32 per cent., and in the general community 0.18 per cent. It is stated, on the authority of three French newspapers, that 20 per cent. of doctors there die of morphinism. But the statement seems almost incredible, even when backed up by the assertion of the secretary of the Association Amicale des Médicins Français that 20 per cent. at least of the members of the profession take morphia or its derivatives almost habitually. Even then there is not the slightest warrant for the application of a similar *dictum* to English doctors, in regard to whom Dr. Jennings has not only failed to prove his case but has made no serious attempt to do so.

